# Comparison of Antimicrobial Susceptibility Patterns of Bacterial Isolates from Blood, Urine, and Lower Respiratory Tract Specimens Between Elderly Patients in Long-Term Care Hospitals and Community-Acquired Infections: A Retrospective Study

**DOI:** 10.3390/antibiotics15060530

**Published:** 2026-05-22

**Authors:** Kye Won Choe, Sumi Yoon, Yong Kwan Lim, Hongkyung Kim, Mi-Kyung Lee, Oh Joo Kweon

**Affiliations:** 1 Department of Laboratory Medicine, Chung-Ang University Gwangmyeong Hospital, Chung-Ang University College of Medicine, 110, Deokan-Ro, Gwangmyeong-Si 14353, Republic of Korea; choekw@cauhs.or.kr (K.W.C.); sumisy@cau.ac.kr (S.Y.); 2 Department of Laboratory Medicine, Chung-Ang University Hospital, Chung-Ang University College of Medicine, 102, Heukseok-ro, Dongjak-gu, Seoul 06973, Republic of Korea; yoorer@naver.com (Y.K.L.); khk.labmed@cauhs.or.kr (H.K.); cpworld@cau.ac.kr (M.-K.L.)

**Keywords:** long-term care hospitals, elderly patients, antimicrobial susceptibility, antimicrobial resistance, minimum inhibitory concentration, bloodstream infections, urinary tract infections, lower respiratory tract infections, emergency department

## Abstract

Background/Objectives: Patients in long-term care hospitals (LTCHs) are at increased risk of harboring antimicrobial-resistant organisms due to frequent healthcare exposure and multiple comorbidities. This retrospective observational study aimed to compare the antimicrobial susceptibility of bacterial isolates from LTCH-onset infections (LTCHIs) with those from community-acquired infections (CAIs) in elderly patients. Methods: This study was conducted at a 700-bed urban tertiary university hospital and included patients aged ≥65 years with positive cultures for bacteremia, lower respiratory tract infections (LRTIs), or urinary tract infections (UTIs) within 48 h of admission. Medical records, including antimicrobial susceptibility test results, were reviewed for a total of 1780 patients and their isolates. Antimicrobial susceptibility patterns were compared between LTCHI and CAI patients. Results: Patients with LTCHI exhibited significantly higher antimicrobial non-susceptibility than those with CAIs across multiple pathogens and antimicrobial classes (*p* < 0.05). In bacteremia, *Staphylococcus aureus*, *Escherichia coli*, and *Klebsiella pneumoniae* from LTCHI cases showed increased non-susceptibility to β-lactams and fluoroquinolones. In LRTIs, *Pseudomonas aeruginosa* and *Acinetobacter baumannii* demonstrated high non-susceptibility to carbapenems (52.9% and 90%, respectively) and aminoglycosides. In UTIs, LTCHI isolates exhibited broader resistance among *Enterobacterales* and *P. aeruginosa*. Notably, the proportion of multidrug-resistant organisms, including carbapenem-resistant *Enterobacterales* (15.4–50.0%) and carbapenem-resistant *Acinetobacter baumannii* (90.5%), was substantially higher in the LTCHI group across all infection sites. Conclusions: Elderly patients with LTCHI are more likely to harbor antimicrobial-resistant pathogens than those with CAIs. Careful consideration of LTCHI origin is therefore essential for empirical antibiotic selection and for strategies aimed at limiting further resistance.

## 1. Introduction

The global increase in the elderly population has led to a corresponding rise in the use of long-term care facilities, particularly in high-income and rapidly aging societies [1,2]. In line with this trend, the Republic of Korea is undergoing rapid population aging; in 2025, individuals aged ≥65 years accounted for approximately 21.2% of the total population (about 10.84 million). Concurrently, the use of long-term care hospitals (LTCHs) has expanded substantially, with the number of LTCH users increasing 7.8-fold, from about 27,000 in 2000 to 210,284 in 2021 [3].

These patients are vulnerable to major infectious diseases, including bacteremia, lower respiratory tract infections (LRTIs), and urinary tract infections (UTIs), due to advanced age and multiple underlying chronic conditions [4,5,6]. Patients in LTCHs frequently undergo repeated transitions between tertiary-care hospitals and long-term care facilities for acute or specialized treatment. Through these transitions, antimicrobial-resistant organisms acquired in tertiary-care hospitals may become colonized and subsequently introduced into and disseminated within long-term care settings [1,2,7]. In addition, clinical and logistical constraints in LTCHs often limit timely antimicrobial susceptibility testing (AST), leading to frequent reliance on empirical antibiotic therapy [8,9,10,11]. Together, these factors contribute to the high prevalence of antimicrobial resistance among bacterial isolates from LTCH patients.

Patients with LTCH-onset infections (LTCHIs) who deteriorate in long-term care or nursing facilities are frequently transferred to tertiary-care hospitals via the emergency department (ED) when acute care needs exceed the resources of the facility. As a result, ED clinicians are often required to initiate empirical antibiotic therapy in this high-risk population before microbiological data become available [8,9,12]. Therefore, understanding antimicrobial susceptibility patterns in LTCHI patients is essential for appropriate empirical antibiotic selection in the ED setting.

Despite the clinical importance of antimicrobial resistance in patients from LTCHs, available studies in the Republic of Korea have primarily focused on specific infection sites or individual pathogens, and comprehensive comparisons across major infection types remain limited. In particular, few studies have simultaneously evaluated antimicrobial resistance patterns in LTCHI involving bacteremia (including sepsis), LRTIs, and UTIs, which represent the most common and clinically significant infections in this population [4,5,6,9,13].

Therefore, the aim of this study was to compare the antimicrobial susceptibility profiles of bacterial isolates from LTCHI patients aged ≥65 years who were transferred to the ED with those from patients aged ≥65 years presenting with community-acquired infections (CAIs). This retrospective study comprehensively analyzed all eligible cases in a real-world clinical setting at a major tertiary-care university hospital in the metropolitan area of Korea, thereby providing practical evidence to support empirical antibiotic selection for elderly patients presenting from LTCHs.

## 2. Results

### 2.1. Baseline Characteristics of Enrolled Patients and Source of Infection

A total of 1780 elderly patients aged ≥65 years were included. Of these, 446, 610, and 1157 had bacteremia, LRTIs, and UTIs, respectively. A total of 399 patients had multiple target infections. Detailed baseline characteristics are provided in Table 1.

No significant differences were observed between the LTCHI and CAI groups in age, diastolic blood pressure, pulse rate, respiratory rate, body temperature, or urine culture positivity, although the proportion of males differed significantly between the groups (Table 1).

In contrast, significant differences were noted in KTAS triage levels, sources of positive cultures (blood, sputum/bronchial washing/suction tip, and multiple sources), systolic blood pressure, and initial mental status. The LTCHI group presented with more severe triage categories, lower systolic blood pressure, higher rates of respiratory specimen positivity and multiple culture sources, and lower blood culture positivity than the CAI group.

### 2.2. Bacteremia

A total of 445 blood specimens yielded 470 bacterial isolates, with *Staphylococcus aureus*, *Pseudomonas aeruginosa*, *Escherichia coli*, and *Klebsiella pneumoniae* being the most frequently identified pathogens. Comparisons of antimicrobial non-susceptibility patterns between LTCHIs and CAIs are summarized in Table 2.

Gram-positive bacteria: For *S. aureus*, the LTCHI group exhibited significantly higher non-susceptibility rates to β-lactams (oxacillin), fluoroquinolones, aminoglycosides, and trimethoprim–sulfamethoxazole compared with the CAI group (*p* < 0.05).

Gram-negative bacteria: No statistically significant differences in antimicrobial susceptibility patterns were observed for *P. aeruginosa* between the two groups. In contrast, *E. coli* isolates from the LTCHI group exhibited significantly higher non-susceptibility to β-lactam/β-lactamase inhibitor combinations, first- to fourth-generation cephalosporins, monobactams, fluoroquinolones, and aminoglycosides compared with the CAI group (*p* < 0.05). Similarly, *K. pneumoniae* isolates from the LTCHI group exhibited significantly higher non-susceptibility rates to β-lactam/β-lactamase inhibitor combinations, cephalosporins, monobactams, carbapenems, fluoroquinolones, aminoglycosides, and trimethoprim–sulfamethoxazole compared with the CAI group (*p* < 0.05).

Specific antimicrobial agents demonstrating significant differences are detailed in Table 2, whereas overall antimicrobial susceptibility, including minimum inhibitory concentration (MIC) values for all blood isolates, is presented in the Supplementary Materials.

### 2.3. Lower Respiratory Tract Infections

From 610 lower respiratory tract specimens, 696 bacterial isolates were recovered. The predominant pathogens included *S. aureus*, *P. aeruginosa*, *Acinetobacter baumannii*, and *Enterobacterales*. Antimicrobial non-susceptibility patterns for these organisms are compared between LTCHIs and CAIs in Table 3.

Gram-positive bacteria: For *S. aureus*, significantly higher non-susceptibility rates to β-lactams (oxacillin), fluoroquinolones, aminoglycosides, trimethoprim–sulfamethoxazole, lincosamides, and macrolides were observed in the LTCHI group compared with the CAI group (*p* < 0.05).

Gram-negative bacteria: In *P. aeruginosa*, the LTCHI group showed significantly higher non-susceptibility rates to β-lactam/β-lactamase inhibitor combinations, fourth-generation cephalosporins, monobactams, carbapenems, fluoroquinolones, and aminoglycosides compared with the CAI group (*p* < 0.05). For *A. baumannii*, non-susceptibility to β-lactam/β-lactamase inhibitor combinations, cephalosporins, carbapenems, fluoroquinolones, aminoglycosides, and trimethoprim–sulfamethoxazole was significantly higher in the LTCHI group than in the CAI group (*p* < 0.05). Among *Enterobacterales*, the LTCHI group exhibited significantly higher non-susceptibility rates to β-lactam/β-lactamase inhibitor combinations, cephalosporins, monobactams, carbapenems, fluoroquinolones, aminoglycosides, trimethoprim–sulfamethoxazole, and tetracyclines compared with the CAI group (*p* < 0.05).

Specific antimicrobial agents demonstrating significant differences are detailed in Table 3, whereas overall antimicrobial susceptibility, including MIC values for all lower respiratory tract isolates, is presented in the Supplementary Materials.

### 2.4. Urinary Tract Infections

A total of 1285 isolates were identified from urine cultures in 1157 patients. The most frequently encountered pathogens included *Escherichia coli*, *Klebsiella pneumoniae*, *Proteus mirabilis*, *Pseudomonas aeruginosa*, *Enterococcus faecium*, and *Enterococcus faecalis*, with most belonging to Gram-negative bacteria. Comparisons of antimicrobial non-susceptibility patterns between the LTCHI and CAI groups are presented in Table 4.

Among *E. coli* isolates, non-susceptibility was significantly more prevalent in the LTCHI group across multiple antimicrobial classes, including penicillins, β-lactam/β-lactamase inhibitor combinations, cephalosporins of various generations, monobactams, carbapenems, fluoroquinolones, and aminoglycosides (*p* < 0.05).

Similarly, *K. pneumoniae* isolates from the LTCHI group showed significantly higher non-susceptibility to β-lactam/β-lactamase inhibitor combinations, cephalosporins, monobactams, carbapenems, fluoroquinolones, aminoglycosides, and trimethoprim–sulfamethoxazole than those from the CAI group (*p* < 0.05).

For *P. mirabilis*, significantly increased non-susceptibility in the LTCHI group was observed mainly for penicillins, cephalosporins, fluoroquinolones, aminoglycosides, and trimethoprim–sulfamethoxazole (*p* < 0.05).

In *P. aeruginosa*, isolates from LTCHI patients showed significantly higher non-susceptibility to antipseudomonal β-lactams, cephalosporins, carbapenems, fluoroquinolones, and aminoglycosides (*p* < 0.05).

No statistically significant differences in non-susceptibility were observed for *E. faecalis* across antimicrobial classes. In contrast, *E. faecium* isolates showed a significantly higher non-susceptibility rate to vancomycin in the LTCHI group (*p* < 0.05).

Antimicrobial classes demonstrating statistically significant differences are summarized in Table 4, while overall antimicrobial susceptibility, including MIC values for all urinary isolates, is presented in the Supplementary Materials.

### 2.5. Multidrug-Resistant Organisms

Comparisons of methicillin-resistant *Staphylococcus aureus* (MRSA), vancomycin-resistant Enterococcus (VRE), carbapenem-resistant *Enterobacterales* (CRE), carbapenem-resistant *Pseudomonas aeruginosa* (CRPA), and carbapenem-resistant *A. baumannii* (CRAB) between the LTCHI and CAI groups are summarized in Table 5.

In blood isolates, the proportion of MRSA was significantly higher in the LTCHI group than in the CAI group (100% vs. 37.9%, *p* = 0.016). CRE was also significantly more frequently identified in the LTCHI group (15.4% vs. 1.22%, *p* = 0.017). Although CRPA was more common in the LTCHI group (66.6% vs. 8.3%), this difference was not statistically significant (*p* = 0.081). No meaningful comparison could be made for VRE due to the absence of cases in the LTCHI group.

In lower respiratory tract isolates, the LTCHI group showed significantly higher proportions of MRSA (36.2% vs. 7.2%, *p* = 0.001), CRE (50.0% vs. 8.6%, *p* < 0.001), CRPA (52.9% vs. 22.9%, *p* = 0.001), and CRAB (90.5% vs. 16.7%, *p* < 0.0001) compared with the CAI group.

In urine isolates, VRE (36.8% vs. 8.4%, *p* = 0.002), CRE (35.4% vs. 8.1%, *p* < 0.001), and CRPA (71.4% vs. 40.0%, *p* = 0.029) were significantly more prevalent in the LTCHI group. MRSA and CRAB were not identified in urine samples from the LTCHI group, precluding statistical comparison.

Overall, the LTCHI group showed consistently higher proportions of multidrug-resistant organisms (MDRO) across most specimen types, with particularly marked differences in carbapenem-resistant organisms.

## 3. Discussion

This retrospective observational study provides clinically relevant evidence on antimicrobial resistance among elderly patients with LTCHI presenting to the ED. By comparing LTCHIs and CAIs across major infection types, our findings highlight higher rates of antimicrobial non-susceptibility in LTCHI cases, underscoring the need for careful empirical antibiotic selection in this high-risk population.

The observed differences in antimicrobial resistance between the LTCHI and CAI groups are consistent with previous studies demonstrating that long-term care facilities serve as major reservoirs of MDROs. Prior investigations have reported higher rates of colonization and infection with resistant pathogens, including MRSA, VRE, and carbapenem-resistant organisms, among patients from long-term care settings compared with those from the community. This pattern has been attributed to cumulative healthcare exposure, frequent antibiotic use, and the increased use of invasive devices in this population [1,2].

When stratified by infection site, distinct patterns emerged. In bloodstream infections, the LTCHI group showed higher resistance rates in *S. aureus*, *E.* coli, and *K. pneumoniae*, whereas no significant differences were observed for *P. aeruginosa*. Similar findings have been reported in prior bacteremia studies, in which *Enterobacterales* from healthcare-associated infections showed higher rates of extended-spectrum β-lactamase production and carbapenem resistance than community-acquired isolates [10,14,15]. The absence of differences in *P. aeruginosa* susceptibility may reflect its intrinsically high baseline resistance and heterogeneous resistance mechanisms [16,17].

In lower respiratory tract infections, resistance differences between the LTCHI and CAI groups were particularly pronounced across major pathogens, including *S. aureus*, *P. aeruginosa*, *A. baumannii*, and *Enterobacterales*. This finding is consistent with previous reports indicating that pneumonia in long-term care facility residents is frequently associated with MDROs, particularly non-fermenting Gram-negative bacilli such as *P. aeruginosa* and *A. baumannii* [1,12,18]. The elevated resistance rates observed in this study likely reflect repeated antimicrobial exposure, chronic colonization of the respiratory tract, and frequent aspiration events in this population.

In urinary tract infections, although resistance patterns were significantly higher in the LTCHI group for most Gram-negative organisms, the differences were less pronounced than those observed in respiratory infections. This is consistent with previous studies showing that, while UTIs in long-term care settings are associated with increased resistance, they remain predominantly caused by *Enterobacterales* with relatively preserved susceptibility compared with respiratory isolates [9,13,19,20]. Nonetheless, the higher resistance rates observed in *E. coli*, *K. pneumoniae*, *P. mirabilis*, and *Pseudomonas aeruginosa* in the LTCHI group underscore the growing impact of healthcare-associated resistance even in traditionally community-dominated infections [14,21].

Importantly, the analysis of MDRO proportions further supports these findings. The LTCHI group showed consistently higher proportions of MRSA, VRE, CRE, CRPA, and CRAB across most specimen types, with particularly marked differences in carbapenem-resistant organisms in respiratory and urinary isolates. This aligns with previous epidemiological data indicating that long-term care facilities play a key role in the dissemination of carbapenem-resistant Gram-negative bacteria, including CRE and carbapenem-resistant non-fermenters [10,13,22,23,24]. The high prevalence of CRAB and CRPA in respiratory specimens in the LTCHI group likely reflects the established association between ventilator- or aspiration-related pneumonia and multidrug-resistant non-fermenting organisms.

Taken together, these findings support the concept of a healthcare-associated resistance gradient, in which patients from long-term care settings exhibit higher antimicrobial resistance than those from the community. Clinically, this highlights the importance of incorporating prior residence in long-term care facilities into empirical antibiotic decision-making, particularly for severe infections such as pneumonia and bacteremia, where delayed appropriate therapy may adversely affect outcomes.

Our study has several limitations. First, the number of patients in the LTCHI group, particularly those with bacteremia, was smaller than that in the CAI group, which may have affected the statistical power of the analysis. With a larger sample size, certain trends, such as higher non-susceptibility rates among *P. aeruginosa* isolates in the LTCHI group across major antipseudomonal antimicrobial classes, might have reached statistical significance. Second, changes in antimicrobial susceptibility testing panels over the study period meant that certain agents were not consistently tested for the same pathogen, depending on the time of isolation. Given the retrospective nature of the study, preservation of isolates was not feasible, and additional experiments could not be performed. Moreover, we performed a large number of statistical comparisons across multiple organism–antibiotic combinations. We did not formally adjust for multiple testing; therefore, some statistically significant findings may reflect chance, and our results should be viewed as exploratory and hypothesis-generating. Third, although respiratory and urinary isolates may partly represent colonization rather than true infection, we attempted to minimize this by restricting inclusion to symptomatic patients undergoing evaluation for suspected infection in the emergency department and by applying quantitative criteria for culture positivity (≥10^3^ CFU/mL for urine cultures, a commonly accepted threshold in complicated or healthcare-associated UTIs, and only “many” growth in sputum cultures after excluding contaminants). Furthermore, because we did not comprehensively review prior healthcare contact history for all patients, we cannot exclude the possibility that some cases classified as community-acquired infections were, in fact, healthcare-associated infections. Fourth, this was a single-center retrospective analysis, which may limit the generalizability of the findings. Fifth, important confounding factors such as prior antimicrobial exposure, comorbidities, functional status, and invasive device use could not be comprehensively adjusted for. Sixth, molecular characterization of resistance mechanisms was not performed, preventing further assessment of specific resistant clones or genotypes. Finally, clinical outcomes such as mortality, length of hospital stay, or appropriateness of empirical antibiotic therapy were not evaluated; therefore, future prospective multicenter studies are warranted.

In conclusion, our findings indicate that elderly patients with LTCHI are substantially more likely to harbor antimicrobial-resistant pathogens than those presenting with CAIs. In particular, LTCHI patients showed markedly higher rates of carbapenem-resistant Gram-negative organisms and MRSA across blood, respiratory, and urinary isolates, with the greatest differences seen in lower respiratory tract infections. These results underscore the importance of considering LTCHI origin when initiating empirical antibiotic therapy in the ED, particularly in this high-risk population. Incorporating knowledge of local resistance patterns and healthcare-associated exposure into early clinical decision-making may improve the appropriateness of initial treatment and contribute to efforts to prevent the further emergence and dissemination of antimicrobial resistance.

## 4. Materials and Methods

### 4.1. Study Design and Subject Selection

This study was a single-center, retrospective observational study conducted at a local university hospital, a 700-bed urban tertiary care center. Elderly symptomatic patients aged ≥65 years who were transferred from LTCHs to the ED, as well as community patients aged ≥65 years presenting to the ED between 1 January 2023 and 31 December 2025, for infectious disease evaluation were screened.

LTCH-onset infections were defined as infections in patients whose route of presentation was recorded as transfer from a long-term care hospital. Community-acquired infections were defined as infections in patients whose route of presentation did not involve a long-term care hospital or any other hospital prior to arrival at our emergency department.

The target infections were bacteremia, including sepsis, UTIs, and LRTIs. Accordingly, patients with positive pathogen cultures from designated specimens (blood, lower respiratory specimens, or urine) collected within 48 h of the ED visit were enrolled; the numbers of patients with LTCH-onset bacteremia, LRTIs, and UTIs were 21, 85, and 83, respectively, and those with community-acquired infections were 424, 525, and 1074, respectively.

Data on culture and antimicrobial susceptibility test results, sex, age, mental status, triage level, and vital signs were collected through review of electronic medical records. Baseline characteristics of the enrolled patients are summarized in Table 1. All data were obtained from electronic medical records and microbiology databases in a de-identified format, and no personally identifiable information was accessible to the investigators. Because of the retrospective design and anonymized data handling, the Institutional Review Board of Chung-Ang University Gwangmyeong Hospital granted an exemption from the requirement for written informed consent (protocol code 2601-300-009; date of approval: 9 February 2026).

### 4.2. Bacterial Culture and Antimicrobial Susceptibility Tests

For blood culture, blood specimens were inoculated into BD BACTEC™ Plus Aerobic/F Culture Vials (BD, Franklin Lakes, NJ, USA) and BACTEC™ Lytic/10 Anaerobic/F Culture Vials (BD). These bottles were incubated in an automated blood culture analyzer (BACTEC™ FX; BD). For the subculture of blood specimens, aerobic isolates were subcultured onto blood agar (BAP), MacConkey (MAC), and chocolate agar, and incubated under standard atmospheric conditions at 35 °C for up to 48 h. Anaerobic isolates were additionally subcultured onto phenylethyl alcohol (PEA) agar and incubated under anaerobic conditions at 35 °C for up to 48 h.

For urine culture, midstream urine specimens were collected in aseptic urine cups and inoculated onto BAP and MAC using a 1:1000 calibrated loop. The inoculated plates were incubated under standard atmospheric conditions at 35 °C for 24 h.

For respiratory culture, sputum, endotracheal aspirates, and suction tip cultures were cultured on BAP and MacConkey agar (MAC). The inoculated plates were incubated under standard atmospheric conditions at 35 °C for up to 48 h.

For all cultures, bacterial identification and antimicrobial susceptibility tests (AST) were performed using MALDI Biotyper Sirius (Bruker Daltonics, Bremen, Germany) and Phoenix M50 (BD, Sparks, MD, USA), respectively. Interpretation of AST results was performed according to the latest Clinical and Laboratory Standards Institute (CLSI) M100 guidelines [25]. All procedures, including manual analyses and interpretations, were conducted by experienced medical technologists and board-certified specialists in laboratory medicine.

For each patient, only the first isolate per organism and specimen type obtained within the first 3 days of presentation was included in the analysis. Repeated cultures yielding the same organism from the same patient during the same hospitalization were not counted separately. Polymicrobial cultures were included only when fewer than three organisms were recovered. In such polymicrobial cultures, if multiple colonies of the same species with different MIC values were present, only the colony with the higher MIC was analyzed; when two different species were isolated, both organisms were included in the organism-specific analyses.

### 4.3. Definitions of Multidrug-Resistant Organisms

MRSA was defined as any *Staphylococcus aureus* isolate resistant to oxacillin or cefoxitin according to CLSI breakpoints. VRE was defined as *Enterococcus faecium* or *Enterococcus faecalis* isolates nonsusceptible to vancomycin. CRE was defined as *Enterobacterales* isolates resistant to at least one carbapenem (imipenem, meropenem, or ertapenem). CRPA and CRAB were defined as *Pseudomonas aeruginosa* or *Acinetobacter baumannii* isolates, respectively, that were resistant to at least one tested carbapenem (imipenem or meropenem).

### 4.4. Statistical Analysis

Categorical variables were compared using the chi-square test or Fisher’s exact test, depending on the sample size of each group. Continuous variables were compared using the Student’s *t*-test or the Kruskal–Wallis test, depending on the normality of the distribution. A *p*-value < 0.05 was considered statistically significant. All analyses were performed using R version 4.3.3 (R Foundation for Statistical Computing, Vienna, Austria), Microsoft Excel 2019 (Microsoft, Redmond, WA, USA), and MedCalc version 23.3.7 (MedCalc Software, Ostend, Belgium).

## Figures and Tables

**Table 1:** Baseline characteristics of aging patients with LTCH-onset and Community-acquired bacteremia, lower respiratory tract, and urinary tract infections.

Variables	LTCHI (*n* = 136)	CAI (*n* = 1644)	*p*-Value
Age, yrs ^1^	79.3 ± 8.9	79.12 ± 8.1	0.823
Male ^2^	70 (51.5)	577 (35.1)	<0.001
KTAS Triage ^2^			<0.001
Level 1; resuscitation	3 (2.2)	49 (3.0)	
Level 2; emergent	62 (45.6)	433 (26.3)	
Level 3; urgent	68 (50.0)	1070 (65.1)	
Level 4; less urgent	3 (2.2)	82 (5.0)	
Level 5; non-urgent	0 (0.0)	10 (0.6)	
Source of Positive Culture ^2^			
Blood ^3^	21 (15.4)	425 (25.9)	0.007
Sputum/bronchial-washing/suction tip ^3^	85 (62.5)	525 (31.9)	<0.001
Urine ^3^	83 (61.0)	1074 (65.3)	0.350
Multiple ^4^	47 (34.6)	353 (21.5)	0.001
Vital sign, initial ^1^			
Systolic BP	122.45 ± 28.46	132.25 ± 31.56	<0.001
Diastolic BP	75.71 ± 17.87	77.55 ± 19.76	0.293
Pulse rate	93.52 ± 18.77	95.40 ± 23.53	0.365
Respiratory rate	20.99 ± 3.14	20.51 ± 4.39	0.209
Body temperature	36.96 (0.77)	37.33 (2.77)	0.128
Mental status, initial ^2^			0.002
Alert	102 (75.0)	1429 (86.9)	
Verbal responsive	16 (11.8)	97 (5.9)	
Painful responsive	17 (12.5)	102 (6.2)	
Unresponsive	1 (0.7)	16 (1.0)	

^1^ Mean ± standard deviation; ^2^ n (%); ^3^ Including cases with multiple positive culture sources; ^4^ Defined as positivity in ≥2 culture sources listed above; Abbreviations: LTCHI, long-term care hospital–onset infections; CAI, community-acquired infections; yrs, years; KTAS, Korean Triage and Acuity Scale; BP, blood pressure.

**Table 2:** Comparison of antimicrobial non-susceptibility patterns of major isolates from blood between long-term care hospital–onset and community-acquired infections.

	*Staphylococcus aureus*			*Pseudomonas aeruginosa*			*Escherichia coli*			*Klebsiella pneumoniae*		
	L (5) ^1^	C (29)	*p*-Value	L (3)	C (12)	*p*-Value	L (9)	C (297)	*p*-Value	L (4)	C (111)	*p*-Value
Penicillin G	100 ^2^	86.2	1.000	-	-	-	-	-	-	-	-	-
Oxacillin	100	37.9	0.016	-	-	-	-	-	-	-	-	-
Ampicillin	-	-	-	-	-	-	100	67.3	0.062	100	100	1.000
Amoxicillin–clavulanate	-	-	-	-	-	-	100	21.1	0.011	50	9.7	0.208
Ampicillin–sulbactam	-	-	-	-	-	-	100	44.4	0.009	100	16.3	0.035
Piperacillin–tazobactam	-	-	-	66.7	16.7	0.154	33.3	3.4	0.004	75	9	0.004
Cefazolin	-	-	-	-	-	-	88.9	48.8	0.036	75	23.4	0.049
Cefuroxime	-	-	-	-	-	-	88.9	43.8	0.013	75	26.1	0.065
Cefoxitin	100	41.2	0.090	-	-	-	11.1	10.1	1.000	50	5.4	0.024
Ceftriaxone	-	-	-	-	-	-	88.9	32.7	0.001	75	19.8	0.032
Ceftazidime	-	-	-	66.7	25	0.242	88.9	26.3	0.000	75	18	0.025
Cefepime	-	-	-	66.7	16.7	0.154	88.9	28.3	0.000	75	18	0.025
Ceftazidime–avibactam	-	-	-	50	14.3	0.417	0	0.6	1.000	0	0	1.000
Ceftolozane–tazobactam	-	-	-	50	14.3	0.417	0	2.3	1.000	0	8.1	1.000
Aztreonam	-	-	-	66.7	25	0.242	88.9	26.3	0.000	75	17.1	0.022
Ertapenem	-	-	-	-	-	-	0	0.3	1.000	50	3.6	0.013
Imipenem	-	-	-	66.7	8.3	0.081	0	0.3	1.000	50	2.7	0.009
Meropenem	-	-	-	66.7	8.3	0.081	0	0.3	1.000	50	2.7	0.009
Ciprofloxacin	100	34.5	0.011	66.7	25	0.242	88.9	51.2	0.038	100	23.4	0.004
Levofloxacin	-	-	-	66.7	16.7	0.154	88.9	41.1	0.005	100	18.9	0.002
Moxifloxacin	100	31	0.007	-	-	-	-	-	-	-	-	-
Amikacin	-	-	-	100	0	0.167	33.3	2.4	0.002	0	0	1.000
Gentamicin	80	20.7	0.019	100	20	0.333	77.8	21.9	0.001	75	2.7	0.000
Tobramycin	-	-	-	50	14.3	0.417	100	24	0.015	100	8.1	0.010
Trimethoprim–sulfamethoxazole	60	10.3	0.029	-	-	-	33.3	34.3	1.000	75	17.1	0.022
Tetracycline	-	-	-	-	-	-	33.3	39.8	1.000	100	21	0.052
Doxycycline	0	0	1.000	-	-	-	-	-	-	-	-	-
Tigecycline	-	-	-	-	-	-	0	1.6	1.000	50	6.1	0.152
Chloramphenicol	NA (*n* = 0)	10	NA	-	-	-	-	-	-	-	-	-
Clindamycin	40	31	1.000	-	-	-	-	-	-	-	-	-
Erythromycin	40	37.9	1.000	-	-	-	-	-	-	-	-	-
Rifampin	0	0	1.000	-	-	-	-	-	-	-	-	-

^1^ Numbers in parentheses in the column headers represent the number of isolates. ^2^ Data are expressed as percentages of isolates categorized as intermediate (I) or resistant (R); not all isolates were tested for every antibiotic due to changes in the susceptibility panel during the data collection period. Abbreviations: L, long-term care hospital–onset infections; C, community-acquired infections; NA, Not Available.

**Table 3:** Comparison of antimicrobial non-susceptibility patterns of major lower respiratory tract isolates between long-term care hospital–onset and community-acquired infections.

	*Staphylococcus aureus*			*Pseudomonas aeruginosa*			*Acinetobacter baumannii*			*Enterobacterales*		
	L (25) ^1^	C (199)	*p*-Value	L (34)	C (191)	*p*-Value	L (20)	C (24)	*p*-Value	L (25)	C (175)	*p*-Value
Penicillin G	100 ^2^	78.4	0.006	-	-	-	-	-	-	-	-	-
Oxacillin	72	36.2	0.001	-	-	-	-	-	-	-	-	-
Ampicillin	-	-	-	-	-	-	-	-	-	92	97.7	0.164
Amoxicillin–clavulanate	-	-	-	-	-	-	-	-	-	66.7	27.4	0.017
Ampicillin–sulbactam	-	-	-	-	-	-	83.3	25	0.012	92.3	46.2	0.002
Piperacillin–tazobactam	-	-	-	44.1	13.1	<0.001	90	20.8	<0.001	36	6.9	<0.001
Cefazolin	-	-	-	-	-	-	-	-	-	95	44	<0.001
Cefuroxime	-	-	-	-	-	-	-	-	-	-	-	-
Cefoxitin	60	37.2	0.186	-	-	-	-	-	-	52	21.7	0.003
Ceftriaxone	-	-	-	-	-	-	-	-	-	68	24	<0.001
Ceftazidime	-	-	-	20.6	14.1	0.310	90	20.8	<0.001	48	18.9	0.003
Cefepime	-	-	-	38.2	15.7	0.004	90	20.8	<0.001	64	19.4	<0.001
Ceftazidime–avibactam	-	-	-	5.9	1.9	0.360	-	-	-	0	1.1	1.000
Ceftolozane–tazobactam	-	-	-	5.9	2.8	0.450	-	-	-	16.7	6.4	0.224
Aztreonam	-	-	-	35.3	18.8	0.041	0	100	NA	52	17.7	<0.001
Ertapenem	-	-	-	-	-	-	-	-	-	20	3.4	0.006
Imipenem	-	-	-	52.9	20.9	<0.001	90	16.7	<0.001	33.3	5.8	0.001
Meropenem	-	-	-	35.3	13.1	0.004	90	16.7	<0.001	16	2.9	0.016
Ciprofloxacin	76	24.6	<0.001	47.1	24.6	0.012	90	25	<0.001	72	30.9	<0.001
Levofloxacin	-	-	-	51.5	29.2	0.016	90	17.4	<0.001	72	28	<0.001
Moxifloxacin	76	23.6	<0.001	-	-	-	-	-	-	-	-	-
Amikacin	-	-	-	26.7	9.7	0.097	75	16.7	<0.001	32	2.3	<0.001
Gentamicin	52	19.1	0.001	26.7	9.7	0.097	100	20.8	<0.001	52	9.1	<0.001
Tobramycin	-	-	-	38.9	3.1	<0.001	62.5	23.1	0.164	58.3	9.5	<0.001
Trimethoprim–sulfamethoxazole	4	2	0.450	-	-	-	85	12.5	<0.001	56	20.6	<0.001
Tetracycline	-	-	-	-	-	-	-	-	-	72.7	19.8	0.001
Doxycycline	0	0	1.000	-	-	-	-	-	-	-	-	-
Tigecycline	-	-	-	-	-	-	-	-	-	20	6.6	0.186
Chloramphenicol	7.7	5	0.537	-	-	-	-	-	-	-	-	-
Clindamycin	60	22.1	<0.001	-	-	-	-	-	-	-	-	-
Erythromycin	60	26.1	0.001	-	-	-	-	-	-	-	-	-
Rifampin	0	0.5	1.000	-	-	-	-	-	-	-	-	-

^1^ Numbers in parentheses in the column headers represent the number of isolates. ^2^ Data are expressed as percentages of isolates categorized as intermediate (I) or resistant (R); not all isolates were tested for every antibiotic due to changes in the susceptibility panel during the data collection period. Abbreviations: L, long-term care hospital–onset infections; C, community-acquired infections.

**Table 4:** Comparison of antimicrobial non-susceptibility patterns of major urinary isolates between long-term care hospital–onset and community-acquired infections.

	*Enterococcus* *faecalis*			*Enterococcus* *faecium*			*Escherichia* *coli*			*Klebsiella* *pneumoniae*			*Proteus* *mirabilis*			*Pseudomonas* *aeruginosa*		
	L (7) ^1^	C (120)	*p*-Value	L (12)	C (57)	*p*-Value	L (36)	C (744)	*p*-Value	L (16)	C (164)	*p*-Value	L (13)	C (57)	*p*-Value	L (21)	C (35)	*p*-Value
PEN	28.6 ^2^	7.5	0.113	100	87.7	0.340	-	-	-	-	-	-	-	-	-	-	-	-
AMP	0	0	1.000	100	87.7	0.340	94.4	67.6	<0.001	100	100	1.000	100	59.6	0.003	-	-	-
AMC	-	-	-	-	-	-	47.6	19	0.004	70	33.3	0.036	33.3	4.2	0.094	-	-	-
SAM	-	-	-	-	-	-	80	49.4	0.032	83.3	29.9	0.015	100	54.5	0.033	-	-	-
TZP	-	-	-	-	-	-	25	3.6	<0.001	68.8	22.6	<0.001	0	1.8	1.000	66.7	22.9	0.002
CZL	-	-	-	-	-	-	77.8	42.7	<0.001	81.2	33.1	<0.001	100	39.3	0.018	-	-	-
CXM	-	-	-	-	-	-	83.3	47.4	<0.001	81.2	36.2	0.001	76.9	43.9	0.062	50	10	0.011
FOX	-	-	-	-	-	-	19.4	11	0.172	50	14.7	0.002	30.8	3.5	0.009	-	-	-
CRO	-	-	-	-	-	-	77.8	36.3	<0.001	81.2	31.1	<0.001	92.3	40.4	0.001	-	-	-
CAZ	-	-	-	-	-	-	75	25.2	<0.001	81.2	30.1	<0.001	53.8	22.8	0.040	57.1	17.1	0.003
FEP	-	-	-	-	-	-	77.8	31.2	<0.001	81.2	29.3	<0.001	69.2	38.6	0.064	71.4	17.1	<0.001
CZA	-	-	-	-	-	-	0	0.5	1.000	0	0	1.000	0	0	1.000	50	10	0.011
CTZ	-	-	-	-	-	-	19	2.4	0.003	50	21.8	0.114	16.7	0	0.200	76.2	40	0.013
ATM	-	-	-	-	-	-	77.8	29.4	<0.001	81.2	29.3	<0.001	46.2	21.1	0.082	61.9	31.4	0.050
ETP	-	-	-	-	-	-	5.6	0.4	0.019	50	9.1	<0.001	0	0	1.000	-	-	-
IPM	-	-	-	-	-	-	5.6	0.5	0.028	43.8	8.5	0.001	-	-	-	66.7	37.1	0.053
MEM	-	-	-	-	-	-	5.6	0.4	0.019	43.8	7.9	<0.001	0	0	1.000	71.4	34.3	0.012
CIP	71.4	49.2	0.440	100	89.5	0.581	83.3	57.3	0.002	68.8	40.2	0.035	100	59.6	0.003	-	-	-
LVX	-	-	-	-	-	-	80.6	52.4	0.001	68.8	35	0.013	100	59.6	0.003	85.7	47.1	0.005
AMK	-	-	-	-	-	-	22.2	4.3	<0.001	12.5	1.2	0.040	76.9	42.1	0.032	52.4	8.6	<0.001
GEN	57.1	38.3	0.432	25	33.9	0.738	44.4	23.8	0.009	56.2	15.2	0.001	100	54.4	0.001	100	7.7	0.007
TOB	-	-	-	-	-	-	71.4	24.1	<0.001	40	23.9	0.271	100	54.2	0.061	66.7	28.6	0.026
GNS																		
STS	14.3	12.5	1.000	0	7.1	1.000	-	-	-	-	-	-	-	-	-	-	-	-
VAN	0	0	1.000	58.3	24.6	0.036	-	-	-	-	-	-	-	-	-	-	-	-
DAP	14.3	16	1.000	91.7	92.9	1.000	-	-	-	-	-	-	-	-	-	-	-	-
LZD	0	3.3	1.000	0	0	1.000	-	-	-	-	-	-	-	-	-	-	-	-
QDP	-	-	-	100.0	0	NA	-	-	-	-	-	-	-	-	-	-	-	-
SXT	-	-	-	-	-	-	41.7	34.1	0.372	68.8	30.5	0.004	84.6	45.6	0.014	-	-	-
TET	-	-	-	-	-	-	47.6	38.3	0.492	40	32.2	0.725	100	100	1.000	-	-	-
NIT	14.3	0.8	0.108	100	98.2	1.000	2.8	1.3	0.409	87.5	61.3	0.054	100	100	1.000	-	-	-
FOS	0	0	1.000	-	-	-	0	1.2	1.000	-	-	-	-	-	-	-	-	-

^1^ Numbers in parentheses in the column headers represent the number of isolates. ^2^ Data are expressed as percentages of isolates categorized as intermediate (I) or resistant (R); not all isolates were tested for every antibiotic due to changes in the susceptibility panel during the data collection period. Abbreviations: L, long-term care hospital–onset infections; C, community-acquired infections; PEN, penicillin G; AMP, ampicillin; AMC, amoxicillin–clavulanate; SAM, ampicillin–sulbactam; TZP, piperacillin–tazobactam; CZL, cefazolin; CXM, cefuroxime; FOX, cefoxitin; CRO, ceftriaxone; CAZ, ceftazidime; FEP, cefepime; CZA, ceftazidime–avibactam; CTZ, ceftolozane–tazobactam; ATM, aztreonam; ETP, ertapenem; IPM, imipenem; MEM, meropenem; CIP, ciprofloxacin; LVX, levofloxacin; AMK, amikacin; GEN, gentamicin; TOB, tobramycin; GNS, gentamicin (synergy); STS, streptomycin (synergy); VAN, vancomycin; DAP, daptomycin; LZD, linezolid; QDP, quinupristin–dalfopristin; SXT, trimethoprim–sulfamethoxazole; TET, tetracycline; NIT, nitrofurantoin; FOS, fosfomycin.

**Table 5:** Comparison of MRSA, VRE, CRE, CRPA, and CRAB Proportions between long-term care hospital–onset and community-acquired infections.

	Blood			LRT			Urine		
	LTCHI	CAI	*p*-Value	LTCHI	CAI	*p*-Value	LTCHI	CAI	*p*-Value
MRSA	100%	37.9%	0.016	36.2%	72.0%	0.001	-	-	-
VRE	0%	14.2%	1	-	-		36.8%	8.4%	0.002
CRE	15.4%	1.22%	0.017	50.0%	8.6%	<0.001	35.4%	8.1%	<0.001
CRPA	66.6%	8.3%	0.081	52.9%	22.9%	0.001	71.4%	40.0%	0.029
CRAB	-	-	-	90.5%	16.7%	<0.0001	-	-	-

Abbreviations: LTCHI, long-term care hospital–onset infections; CAI, community-acquired infections; MRSA, Methicillin-Resistant *Staphylococcus aureus*; VRE, Vancomycin-Resistant Enterococcus; CRE, Carbapenem-Resistant *Enterobacterales*; CRPA, Carbapenem-Resistant *Pseudomonas aeruginosa*; CRAB, Carbapenem-Resistant *Acinetobacter baumannii*.

## Data Availability

The data presented in this study are available on request from the corresponding author due to ethical considerations.

## Supplementary Materials

The following supporting information can be downloaded at: https://www.mdpi.com/article/10.3390/antibiotics15060530/s1, Table S1: Antimicrobial susceptibility and minimum inhibitory concentration (MIC) values of all bacterial isolates from blood, lower respiratory tract, and urine specimens.

## Abbreviations

The following abbreviations are used in this manuscript:

LTCH	Long-term care hospital
AST	Antimicrobial susceptibility testing
LRTI	Lower respiratory tract infection
UTI	Urinary tract infection
ED	Emergency department
LTCHI	Long-term care hospital-onset infection
CAI	Community-acquired infection
KTAS	Korean triage and acuity scale
MIC	Minimum inhibitory concentration
MRSA	Methicillin-resistant *Staphylococcus aureus*
VRE	Vancomycin-resistant Enterococcus
CRE	Carbapenem-resistant *Enterobacterales*
CRPA	Carbapenem-resistant *Pseudomonas aeruginosa*
CRAB	Carbapenem-resistant *Acinetobacter baumannii*
MDRO	Multidrug-resistant organisms
CLSI	Clinical and Laboratory Standards Institute
